# Diversity of Treatments in Overcoming Morphophysiological Dormancy of *Paeonia peregrina* Mill. Seeds

**DOI:** 10.3390/plants13162178

**Published:** 2024-08-06

**Authors:** Željana Prijić, Sara Mikić, Jovan Peškanov, Xiuxin Zhang, Lili Guo, Ana Dragumilo, Vladimir Filipović, Goran Anačkov, Tatjana Marković

**Affiliations:** 1 Institute for Medicinal Plants Research “D Josif Pančić” Belgrade, Tadeuša Košćuška 1, 11000 Belgrade, Serbia; smikic@mocbilja.rs (S.M.); adragumilo@mocbilja.rs (A.D.); vfilipovic@mocbilja.rs (V.F.); tmarkovic@mocbilja.rs (T.M.); 2 Department of Biology and Ecology, Faculty of Sciences, University of Novi Sad, Trg Dositeja Obradovića 3, 21000 Novi Sad, Serbia; jovan.peskanov@dbe.uns.ac.rs (J.P.); goran.anackov@dbe.uns.ac.rs (G.A.); 3 Key Laboratory of Biology and Genetic Improvement of Horticultural Crops, Institute of Vegetables and Flowers, Chinese Academy of Agricultural Sciences, Ministry Agriculture and Rural Affairs, Beijing 100081, China; zhangxiuxin@caas.cn; 4 College of Agriculture/Tree Peony, Henan University of Science and Technology, Luoyang 471023, China; guolili@haust.edu.cn

**Keywords:** herbaceous peony, protected species, germination stages, seed coat rupture, epicotyl dormancy release, hypocotyl dormancy release, imbibition, GA_3_, nursery plant production

## Abstract

*Paeonia peregrina* Mill. is a protected, herbaceous species native to Southeastern Europe and Turkey. Due to its vulnerability, it has to be protected both in its natural habitats and through cultivation. Peonies are known to have a low potential for natural regeneration due to their seed dormancy, low germination rate, and long germination period. In this study, treatments with gibberellic acid (GA_3_ 150, 200, 250, 300, and 350 mg L^−1^ GA_3_) and warm (at 20/16 °C day/night regime) and cold stratification (at 4 °C) were used to accelerate dormancy release and increase the germination rate. The seeds of *P. peregrina* from four natural habitats in Serbia and the Institute’s collection were collected and analyzed. They showed an underdeveloped embryo that needs to grow inside the seed before it can germinate. The application of GA_3_ accelerated each stage of germination (seed coat rapturing, hypocotyl dormancy release, and epicotyl dormancy release) for approximately 10 days compared to the control. It was also found that two-day imbibition with 200 mg L^−1^ GA_3_ significantly accelerated and equalized germination. Higher GA_3_ concentrations had a more pronounced impact on each stage but also resulted in greater seed infection after the seed coat rupture, elongated and weak seedlings, while lower concentrations did not result in obtaining uniform seedlings. There were no significant differences observed between localities. Restoring *P. peregrina* through seeds and nursery-produced plants is crucial for conserving the genetic diversity of the tested species.

## 1. Introduction

The peony family (*Paeoniaceae*) includes only the genus *Paeonia* L., with 34 species native to the Northern Hemisphere, of which 25 are herbaceous and 9 are wooden [1]. The genus has been highly appreciated worldwide because of its remarkable qualities, including its high ornamental [2,3], edible [3], and medicinal [4,5] values. Although peonies have been highly respected throughout history, wild species are becoming increasingly rare and/or endangered in their native habitats. As a result, most wild peony species are protected by the law in many countries, including China [6], Romania [2], Russia [7], Serbia [8], and others.

Since peonies are threatened, it is necessary to preserve the genetic diversity of the species. As peony seeds are an important source of genetic diversity, they should be used for the conservation and regeneration of natural populations. In addition, cultivating peonies from seeds can serve to establish new populations in areas where they have become locally extinct. However, a major obstacle to their germination is the well-known double dormancy of their seeds [9,10,11], which is, according to the latest classification, named morphophysiological seed dormancy [12]. Seed dormancy is a biological mechanism that ensures that seeds germinate at the right time and under suitable conditions [13], as it is controlled by a combination of genetic; hormonal (abscisic acid—ABA and gibberellins (GA); and external environmental factors (temperature, humidity, etc.), as well as physiological, biochemical, and molecular regulatory processes.

The temperature requirements of peonies are adapted to the temperate regions of the Northern Hemisphere [14,15], so the adaptation has led to the development of specific growth patterns to synchronize with their annual growth cycle [16]. Therefore, the germination protocol for peonies is complex and requires the separate breaking of dormancy: warm stratification for embryo growth and radicle protrusion, followed by cold stratification for epicotyl growth [17]. In some species, GA_3_ can replace temperature requirements (cold and/or warm) to overcome dormancy [18]. In addition, germination is also slowed by an underdeveloped embryo. The underdeveloped (rudimentary) embryo is a characteristic of many temperate species, including the Paeoniaceae family, and is characterized by the embryo growing to a critical size inside the seed before it germinates [13,19,20]. Therefore, peonies can take up to two years to germinate [11,21]. Herbaceous peony species take up to 10 years to grow from seed to flowering plant [10].

Since the germination of peony seeds is a long-term process, the determination of seed characteristics and pre-treatments that have a positive effect on germination is of particular importance for both the cultivation and conservation of the tested species [14]. Various pre-treatments (physical and/or chemical) can be used to improve the germination rate of peony seeds [22]. Treatments such as presoaking, chilling, and the use of gibberellic acid have been shown to have a positive effect on peony seed germination [14]. Imbibition or water soaking is considered the first phase of seed germination and is crucial for plant growth and development. Water soaking has been reported to improve seed germination of both tree and herbaceous peonies by softening and moistening their hard coat [23], allowing water to penetrate the seed and initiating various enzyme and metabolic reactions that lead to seed germination [14].

The germination performance and growth of many plant species under both normal and stress conditions could be effectively improved by optimizing the concentrations of plant growth hormones such as ABA and GA_3_. The ratio between the two mentioned hormones plays a key role in the dormancy process [24]. A high ABA:GA_3_ ratio maintains dormancy, while a low ABA:GA_3_ ratio causes the release of dormancy [25,26].

Unfortunately, there is little scientific data regarding the growing conditions of herbaceous peonies from seeds. The majority of research has focused on woody peony species [17,18,27,28], and only a few have referred to herbaceous ones [9,21,29,30]. Despite the various methods for breaking seed dormancy described in the available literature, to the best of our knowledge, there still remains a notable absence of a proper methodology procedure for producing nursery plants from dormant *P. peregrina* seeds.

The aim of this study was to determine the requirements for germination of the hypocotyl (root) and epicotyl (shoot) of *P. peregrina* and to observe the effects of different GA_3_ concentrations and cold stratification on the release of dormancy in order to develop a method for shortening the germination period and obtaining uniform nursery plants in the shortest possible time.

## 2. Results

### 2.1. Seed Characteristics

Seeds of *P. peregrina* are large, ellipsoidal, and shiny-black, with a smooth seed surface. Depending on their origin, their average mass ranges in the interval from 0.13 to 0.19 g. The seeds from nature do not significantly differ between themselves, but the seeds from the Institute’s collection were, on average, 36% heavier and significantly differed from the seeds from all other studied localities (Table 1). Seed lengths and widths varied, on average, 7.76–7.89 and 5.1–6.2 mm, respectively. The seeds from the Institute’s collection were approximately 15% larger compared to the seeds from all other localities. The incidence of undeveloped seeds in all samples ranged in the interval 2.9–4.5% (Table 1).

### 2.2. Imbibition

The results of the preliminary research indicate that there was no significant difference in the imbibition rate of *P. peregrina* seeds between the three tested GA_3_ concentrations (200, 300, and 400 mg L^−1^) compared to the control. However, the imbibition period showed a significant difference. Within the three days of imbibition, the amount of absorbed water was more than 33% of the initial seed mass; approximately 80% of the water was absorbed in the first 24 h and less than 5% by the end of the third day (Table 2). Based on the obtained results, a two-day imbibition period was determined for further experiments with all *P. peregrina* seed samples.

### 2.3. Seed Dormancy Release

Our research findings revealed that the seed origin (localities) did not show a significant effect on germination. On the other hand, the application of GA_3_ accelerated each germination stage (seed coat rapturing, hypocotyl dormancy release, and epicotyl dormancy release) and made them more equal.

**Figure 1:**
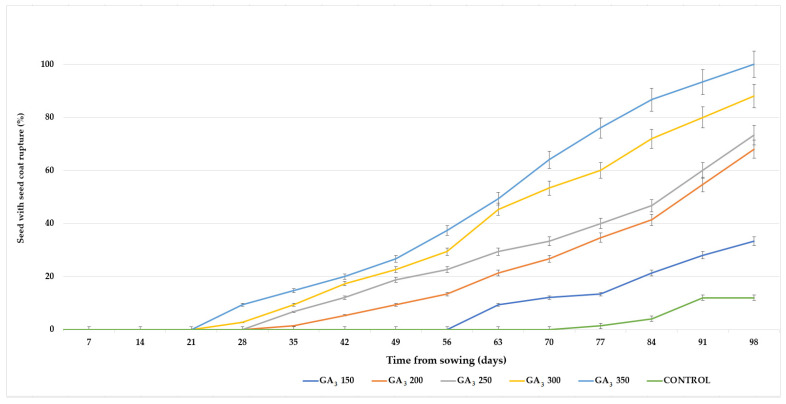
Incidence of *P. peregrina* seeds with a ruptured coat (%), depending on the applied GA_3_ concentration. Error bars indicate the standard error (n = 3).

**Figure 2:**
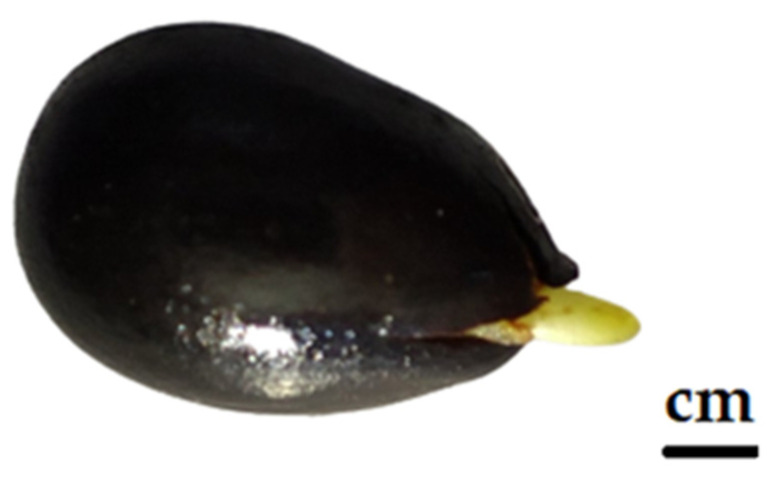
*P. peregrina* seed with a radicle.

The incidence of the stage defined as a ruptured seed coat depended on the applied GA_3_ concentrations (Figure 1). The impact of GA_3_ application on accelerating seed germination was detected. Each 50 mg L^−1^ increase in GA_3_ concentration accelerated seed germination by approximately 10 days. The 300 and 350 mg L^−1^ GA_3_ initiated coat rapturing after a month. In control C1 (seeds immersed in sterile distilled water—SDW), the seed coats started to rupture after 70 days. In the treatment with 350 mg L^−1^ GA_3_, on average, 100% of the seeds had a ruptured coat after 100 days, while control C1 reached only 12%. In control C2 (seeds without imbibition), none of the seeds had a ruptured coat in the first 100 days. The 350 mg L^−1^ GA_3_ concentration was 65.3% and 88.0% more efficient than the lowest GA_3_ concentration (150 mg L^−1^ GA_3_) and C1, respectively (Figure 1). Two weeks after initiated coat rapturing the radicle begin to emerge (Figure 2).

**Figure 3:**
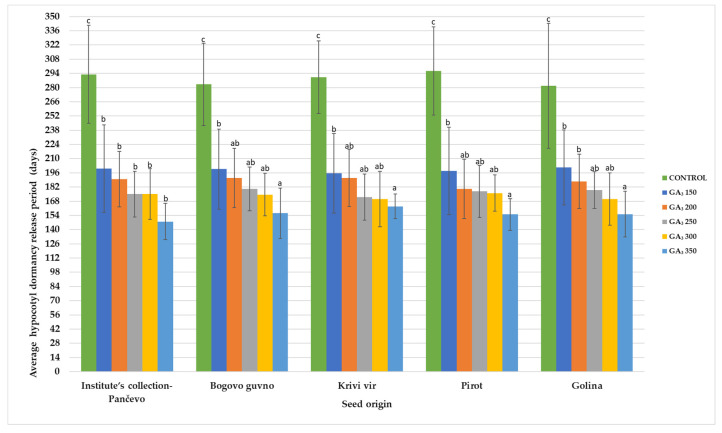
Period required for hypocotyl dormancy release in *P. peregrina* seeds of various origins, depending on the applied GA_3_ concentration. Differences were evaluated by two-way analysis of variance (ANOVA) completed with Duncan’s test (*p* ≤ 0.05). Different letters (a–c) indicate significant differences (*p* ≤ 0.05). Error bars indicate standard deviations (n = 3).

The time required for the occurrence of the hypocotyl dormancy release of *P. peregrina* seeds from various localities in Serbia in treatments with different GA_3_ concentrations is presented in Figure 3. It covers the period of time from the beginning of the experiment until the seeds reach a root length of 30 mm and is presented on a weekly basis.

The results of our research show that more than 150 days (21 week) are needed to obtain seeds with roots up to 30 mm. The difference in the time required for the soaked seed to reach a 30 mm root is, on average, 11 days per treatment, and the difference between the treatment with the lowest tested GA_3_ treatment (150 mg L^−1^ GA_3_) and the control C1 (seeds were imbibed in SDW for two days) is more than 90 days (13 weeks).

**Figure 4:**
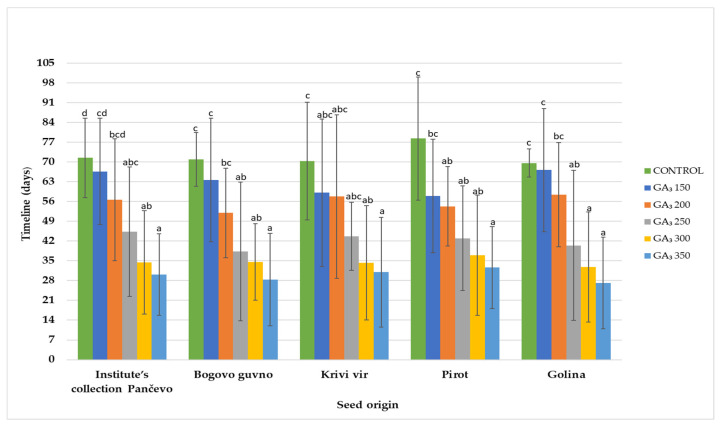
Period required for epicotyl dormancy release. The time presented on the *Y*-axis corresponds to the period from when the seeds reach a root size of 30 mm until the epicotyl reaches a length of 2 mm. Differences were evaluated by two-way analysis of variance (ANOVA) completed with Duncan’s test (*p* ≤ 0.05). Different letters (a–d) indicate significant differences (*p* ≤ 0.05). Error bars indicate standard deviations (n = 3).

The time required for the occurrence of epicotyl dormancy release of *P. peregrina* seeds from various localities in Serbia in treatments with different GA_3_ concentrations is presented in Figure 4. It covers the period on lower temperatures (4 °C) (from the time when seeds reach root sizes up to 30 mm until the epicotyl reaches a length of 2 mm).

For epicotyl development from the seeds with a 30 mm root, a minimum of one month on average at a temperature of 4 °C (treatment with 350 mg L^−1^ GA_3_) was required. In comparison, the control treatment extended this period to over two months, totaling 72 days on average. There was a notable difference of 9 ± 2.8 days between the treatments (Figure 4).

### 2.4. Embryo to Endosperm Ratio (E:S Ratio)

**Figure 5:**
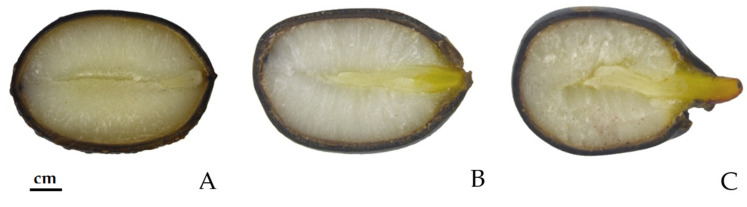
Cross-sectional view of the *Paeonia peregrina* embryo developmental stages: (**A**) fresh seed; (**B**) seed coat rupture; (**C**) radicle protrusion.

The results of the ratio between the lengths of the embryo (E) and endosperm (S), presented in Table 3, indicate that the embryos must elongate before radicle emergence (Figure 5), thus confirming morphological dormancy in *P. peregrina* seeds. In fresh *P*. *peregrina* seeds, the embryo length was, on average, about 40% smaller than in seeds with a ruptured coat (Figure 5) and three times smaller than in seeds with an emerged radicle (Figure 2 and Figure 5 and Table 3).

### 2.5. Field Experiment

**Figure 6:**
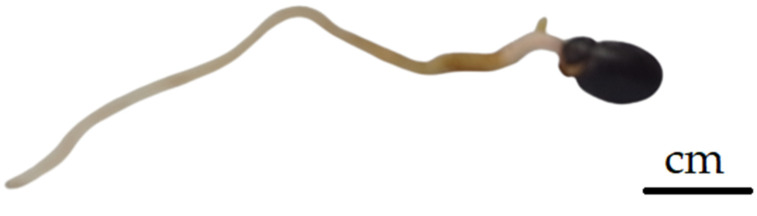
*P. peregrina* seed with epicotyl length up to 2 mm (i.e., morphophysiological seed dormancy is released).

Seeds of *P. peregrina* which epicotyl length reached 2 mm (Figure 6) were transferred from Petri dishes to plastic pots, and the emergence of seedlings above the surface were monitored. As the observations were set to be on every 7th day, differences in the emergence of seedlings between the treatments and localities were not noticed.

The data on the initial growth of *P. peregrina* seedlings, measured on the 21st day (i.e., three weeks following the seedlings emergence), were as follows: the lowest height of 10.1 ± 1.1 cm was observed in the control, while, in the treatments with GA_3_ 150, 200, 250, 300, and 350 mg L^−1^ GA_3_, the heights were 12.5 ± 1.04, 13.9 ± 1.01, 13.8 ± 1.01, 14.1 ± 1.02, and 14.5 ± 1.07 cm, respectively. Statistical analyses of these and other measurements associated with monitoring nursery plant development were not performed, as they were not comparable. The plants did not grow in the same environmental conditions, since the pots were transferred outdoors in a wide interval: from mid-February for the GA_3_ treatments and from mid-August for the control pots.

During the two-year pot production of nursery plants established outdoors, no signs of disease were observed. Regardless of the origin or applied GA_3_ concentrations, the average rate of the regeneration of nursery plats was observed by the end of spring 2023 and 2024, and it was 96%. Spring 2024 was considered the beginning of the first vegetation of nursery plants in the control, and the rate of their survival during the winter period was 100%.

## 3. Discussion

To date, investigations concerning wild herbaceous peonies have mainly focused on geographical distribution [9,31,32], their quantification, habitat conservation [33], and/or chemical composition [5], whereas their cultivation has remained relatively understudied. Peonies are known to have a low germination rate and irregular germination, resulting in a limited natural regeneration capacity [1,14]. In addition, the dispersal of seeds by animals in their native habitats is low [34,35]. If we consider anthropogenic influences on natural habitats and climate change, it is unsurprising that these species face endangered status.

Five herbaceous peony species are native to the Republic of Serbia; the most prevalent is *P*. *peregrina*; thus, research has been conducted on the mentioned species, since the allowed number of seeds collected from nature is limited. The populations of the tested species in Eastern Serbia are mostly at a satisfactory level [31,36], considering that it is protected by law. In this study, it was also confirmed that a sufficient number of seeds was produced per plant at each locality. The research by Sehgal et al. [37] indicated that seed productivity might be decreased if the early stage of seed maturity is affected by drought. Although diseases can also reduce productivity, the appearance of fungi from the *Alternaria*, *Fusarium*, and *Penicillium* genera on herbaceous peonies did not affect seed production [38].

The shape and color of the studied seeds of *P. peregrina* were in agreement with earlier reports [1,39,40,41]. Seed mass from the natural habitats were also within the intervals reported in the literature [1,39,40]. Although seed mass could be influenced by the year, age of the plant, and position of the plant in the environment [1], in this study, the masses of the seeds from natural stands did not significantly differ between themselves but were significantly lighter than the seeds from the Institute’s collection. Considering the more favorable growing conditions and lack of competitive pressures, it was expected that the seeds from the collection would be larger and heavier. The seed size (length x width) in this study was in agreement with previous reports [1,39,40,41]. As peony seeds are considered relatively large, their germination process relies less on light [9], and in this study, they were kept to germinate in darkness. Regardless of the fact that the seeds from the Institute’s collection were heavier and larger than those from natural habitats, in this study, a significant increase in their germination rate was not observed, as suggested in previous reports [42,43,44].

A hard seed coat (but water-permeable) prevents water uptake effectively, thus slowing seed germination [12]. Peony seeds have such a coat, which needs to be softened and moistened to enhance germination [23]. From the perspective of seed biology, germination begins with imbibition [14], since various enzyme and metabolic reactions occur in it, resulting in germination [41]. The length of the imbibition period varies among peony species in the interval of 1 to 3 days [14], and for seeds of *P*. *peregrina*, it was suggested for 2 to 3 days [40]. Three days is more suitable for seeds with a lower moisture content, as, in such seeds, the imbibition starts slowly [40]. As the imbibition rate in our preliminary study was below 5% on the 3rd day, therefore, in this study, two days for the imbibition period was applied. Zhang et al. [14] recommended using warm water for imbibition, as it improves the germination of tree and herbaceous peony seeds.

The germination of peony seeds could also be described as the process of releasing dormancy [45].

According to the seed dormancy classification [12,13,19,46], peony seeds have morphophysiological dormancy (MPD) if they have a rudimentary embryo and require a dormancy-breaking treatment to enable germination, such as exposure to high and/or low temperatures. For embryo growth inside the seed and radicle protrusion, peonies need moist warm (≥15 °C), and for epicotyl growth, peonies needs moist cold [14]. Cold stratification increases enzyme activities, causing substance transformation [30]. In our study, the embryo of *P*. *peregrina* elongates inside the seed to more than three times its initial length in order to germinate (Figure 2 and Figure 3) and can be considered underdeveloped. The same proportion of embryo elongation is also required for the germination of woody peony *P*. *ostii* [18].

The seeds of *P*. *peregrina* had linear embryos, and the fresh seeds had an embryo L (length):W (width) ratio of 2.95 ± 0.01, which is consistent with the results of studies on *P*. *brownii* [45]. The critical embryo length for germination is the length of the embryo at the time when the seed coat divides but before the radicle appears [47]; thus, the critical E:S ratio is determined at the mentioned stage. The results of our studies on *P*. *peregrina’s* E:S ratio are consistent with the studies on the other peony species, *P*. *ostii* [16].

Temperature is considered one of the most important environmental factors that influences the growth and development of many plant species [48]. Temperature requirements of peonies vary during their annual growth cycle, and they also vary between different species and even between cultivars of the same species [14]. During the stratification procedure, the temperatures should be similar to the corresponding natural environment, which should range for warm stratification between 15 °C and 25 °C over 1 to 3 months or until the hypocotyl reaches a species-specific length [14]. In this study, the warm stratification continued until hypocotyl reached a length of 30 mm. Previous reports on cold stratification [49] have shown that temperatures between −7 °C and 7 °C, in a range of 480 to 900 h (i.e., 20–38 days), overcome epicotyl dormancy in most herbaceous peonies, which is not consistent with our studies on *P. peregrina*, where it took almost twice as much time if only a low temperature treatment was used. Additionally, peony plants require low winter temperatures to break underground bud dormancy as well, enabling shoot emergence in the spring [50].

The trend of climate change and high summer temperatures can cause stress for many plant species [51], including peonies, as they are not adapted to a climate with high temperatures [52]. As winter temperatures increase as well, the question arises whether there will be a sufficient sum of low temperatures for peony germination in the future, since increasing winter temperatures affect seed germination and dormancy release [13], prolonging the germination period and increasing the share of abnormal seedlings [53].

An analysis of temperature data from 1888 to 2006 across 15 stations in Serbia revealed a notable increase in air temperatures, particularly in minimum temperatures during winter [54]. Projections suggest that the average global temperature could rise between 0.3 and 4.5 °C by 2100 compared to the period from 1986 to 2005 [55]. This temperature shift may cause the migration of plant species to higher altitudes where they were unable to survive in the past [56] and/or cause them to move northerly in the Northern Hemisphere from their existing habitats [57]. This can be a problem for species such as peonies with a low dispersal of seeds in nature [34,35]. Seeds with epicotyl dormancy show insensitivity to cold treatment until the root reaches a specific length [51,52,58,59]. If, in the period of reaching a specific root length, the temperature does not drop low enough, the root grows and forms lateral roots instead of the shoot [58], which results in a low percentage of seedlings [14]. If the root length is more than 3 cm under normal growth conditions, the germination rate can be higher than 90%. Otherwise, it remains below 50% [14].

The impact of growth hormones applied in various concentrations on the acceleration of germination in different peony species has already been confirmed [9,14,60]. In this study, a two-day imbibition with 200 mg L^−1^ GA_3_ significantly accelerated and equalized the germination rate, which is in agreement with reports on woody peonies. Chilling treatments combined with 100 or 200 mg L^−1^ GA_3_ have proven beneficial for both the germination and growth of *P. ostii* hybrid ‘Feng Dan’ seedlings [17]. In seeds of *Paeonia suffruticosa* Andrews, hypocotyl was differentiated in untreated seedlings, but the dormancy of the epicotyl was not released, and the shoot was not formed. On the other hand, seedlings stored at 5 °C for 8 weeks or treated with GA_3_ formed shoots [61]. The 200 mg L^−1^ GA_3_ increased the germination rate in the mentioned species more than low temperatures [23]. The impact of GA_3_ and chilling on the germination and seedling growth of *P. rockii* hybrids was also observed [60].

Due to the different GA_3_ treatments and the inconsistent germination rates, variations in the length of the vegetation period were observed in this research. Therefore, one of the research objectives was to evaluate the effects of GA_3_ seed treatments on juvenile plants, as well as the percentage of plant regeneration, as little information is available on the juvenile stage of peony species.

## 4. Materials and Methods

This study included laboratory and field experiments, both conducted with seeds of *Paeonia peregrina* Mill.

### 4.1. Seed Origin

**Figure 7:**
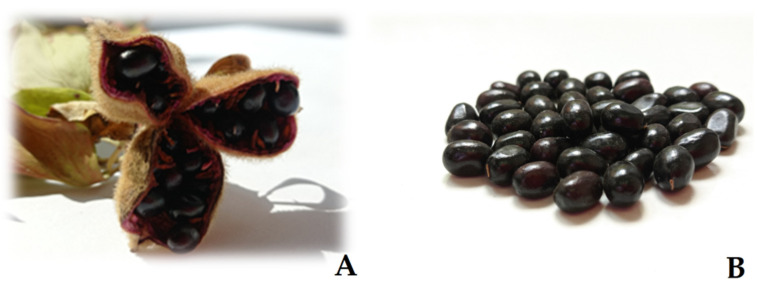
*Paeonia peregrina*: (**A**) mature pods with seeds; (**B**) mature seeds.

**Figure 8:**
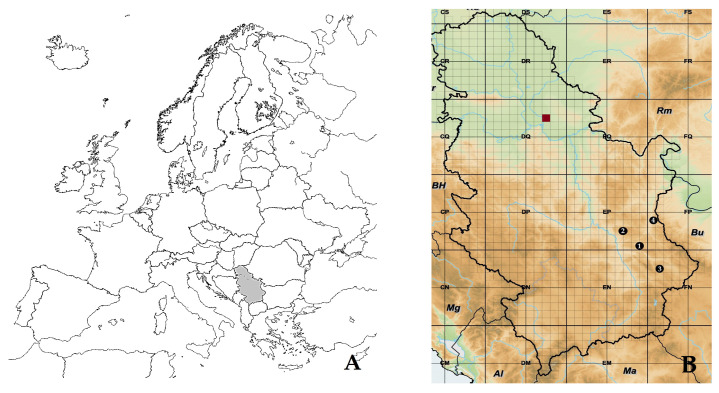
(**A**) Position of the Republic of Serbia on the European continent. (**B**) Localities of *Paeonia peregrina* seed collection: (1) Bogovo guvno; (2) Krivi vir; (3) Pirot; (4) Golina; (red rectangle) Institute’s collection, Pančevo.

*P. peregrina* mature pods with seeds (Figure 7) were manually harvested from four natural stands in Serbia (Figure 8 and Table 4) in August 2021.

About 30 plants per population were harvested, with up to one-third of the seed pods coming from a single plant (approximately 300 seeds per locality). Permission for wild seed collection was granted by the Ministry of Environmental Protection of the Republic of Serbia (No. 353-01-1467/2021-04, issued on 21 May 2021). Also, mature seed pods (approximately 650 seeds) were harvested from plants deriving from the Institute’s collection (Figure 8 and Table 4).

### 4.2. Laboratory Experiment

#### 4.2.1. Seed Measurements

The mass of each seed (300 seeds) per locality was weighed on an analytical balance (Kern and Sohn ABJ 220–4NM, 0.0001 Readability). The collected data were used to calculate the average mass of seeds per each locality, as well as the variation from the average. Mean mass of the seeds per locality and the deviation from the mean between the different localities were also calculated. Additionally, the seeds’ mass was recalculated based on their absolute dry mass. The ratio of undeveloped to fully developed seeds was also estimated. Undeveloped seeds were not included in further experiments.

#### 4.2.2. Seed Surface Disinfection

Since peony seed germination is a long-term process, special attention was paid to the methods for disinfection of its surface prior to experiment. After preliminary testing of different methods [62], the following one was used: the seeds were first immersed in 4% sodium hypochlorite solution (NaClO) for 5 min, rinsed in SDW for 2 min and dried on sterile filter paper, then immersed in 70% ethanol for 5 min and rinsed in SDW for 2 min, and dried and sprayed with fungicide Metconazole in concentrations of 2 mL L^−1^.

#### 4.2.3. Imbibition

The influence of different GA_3_ concentrations (200, 300, and 400 mg L^−1^ GA_3_) in comparison to the control (distilled water) on the rate of imbibition of *P. peregrina* seeds was estimated only on seeds deriving from the Institute’s collection. For determination of the imbibition rate, 30 seeds per treatment were measured individually prior to the imbibition period and 24, 48, and 72 h after this period, as determined in our previous study [36]. During the imbibition period, the seeds were kept at 22 °C, and prior to each measurement, they were dried with sterile paper. The imbibition rate was calculated using the following formula:

$$\mathrm{Imbibition}\;\mathrm{rate}=\frac{Imbibed\;weight-Initial\;weight}{Imbibed\;weight}\;\times \;100$$

Depending on the outcome, the same imbibition period will be applied to all seed samples.

#### 4.2.4. Seed Dormancy Release

In this study, seed dormancy release was examined throughout two stages, hypocotyl dormancy release and epicotyl dormancy release.

Hypocotyl dormancy release. The experiment with *P. peregrina* seeds included three replicates for each treatment and locality, with fifteen seeds per replicate. To determine the best amount of gibberellic acid (GA_3_) (90+%purity, Sigma-Aldrich Chemical Co., Ltd., St. Louis, MO, USA) to break seed dormancy, the following concentrations were tested: 150, 200, 250, 300, and 350 mg L^−1^ GA_3_. The seeds were soaked in warm sterile distilled water (SDW) with a certain concentration of GA_3_ for 48 h. Two control treatments were set up: control C1 involved an imbibition period of 48 h in SDW, whereas C2 had no imbibition period. All seeds were placed on sterilized and moistened double-layer filter paper in covered 90 mm diameter Petri dishes, which were placed in the seed germination chamber, in darkness, in relative humidity of approximately 65% at a 12 h regime of 20/16 °C. The observations of the seeds were conducted weekly. This phase was considered complete when first treatment with GA_3_ reached 100% for hypocotyl dormancy release in all repetitions; the obtained results were expressed as a mean of three replicates (±SD). At this point, the length of the root was approximately ≥ 30 mm [13].

Epicotyl dormancy release. All seeds with root lengths ≥ 30 mm were transferred to new covered Petri dishes with a diameter of 90 mm on double layers of Philter paper moistened with SDW, which were placed in the seed germination chamber, in darkness, in a relative humidity of approximately 60% and at a temperature of 4 °C to promote epicotyl growth [13]. This phase was also monitored weekly, and it was considered complete when epicotyl reached a length of 2 mm, considering that morphophysiological seed dormancy was released.

#### 4.2.5. Embryo and Seed Ratio (E:S Ratio)

The ratio of embryo (E) to endosperm (S) provides insights into seed quality and its germination potential [63]. The measurements of embryonic development were performed on seeds of *P. peregrina* from the Institute’s collections at the following seed stages: fresh seed, seed with a ruptured seed coat, and seed with an emerged radicle. The seeds were cut in half and observed under a binocular microscope (Leica Microsystems GmbH, Wetzlar, Germany) at 6.3× magnification. The Leica DFC290 HD digital microscope camera (with Leica LAS v4.11 software) was used to photograph the embryos. The lengths of the embryos were analyzed using ImageJ image analysis software 1.41o (National Institutes of Health, Bethesda, MA, USA).

### 4.3. Field Experiment

This experiment was conducted with seeds of *P. peregrina* which epicotyl length reached 2 mm (i.e., morphophysiological seed dormancy was released). Such seeds were transferred from Petri dishes to plastic pots filled with 250 mL of peat-based substrate (Gramoflor Green), in which they were sown to a depth of 1 cm. The first two weeks, the pots were kept in a “Grow box” (dimensions 300 × 150 × 200 cm) under regular watering and lighting regimens in a day/night interval of 12–12 h (Biolux 36W fluorescent tubes), and then, the pots were transferred to open field conditions. The time required for the seedlings to emerge above the ground and their heights by the end of the 3rd week following the emergence were measured. The health status of nursery plants was monitored during a two-year nursery plant production period. Also, the regeneration of nursery plants following the first winter was recorded at the beginning of their vegetation (spring), and the obtained results were expressed in percentages.

### 4.4. Statistical Analysis

All statistical analyses were performed with IBM SPSS Statistics Version 25.0 for Windows. A two-way ANOVA was performed to test the effect of the treatments (5 different GA_3_ concentrations); the different locations (Bogovo guvno, Krivi vir, Pirot, Golina, and Pančevo); and their interactions. Statistical significance was registered only for the effects of the treatments. In addition, a one-way ANOVA was applied to the data of a number of days at different GA_3_ concentrations and to the E:S ratio. Multiple comparisons were performed using Duncan’s test to detect significant differences between the arithmetic means of the number of days in the same location and of the E:S ratio (*p* < 0.05)

## 5. Conclusions

In the presented study, it was determined that the seeds of *Paeonia peregrina* Mill. contain an underdeveloped embryo with deep, simple, epicotyl morphophysiological dormancy. In the previous period, there was limited knowledge of the effective methods to break dormancy and establish the cultivation of this valuable plant species from seeds. However, through a three-year study conducted in Serbia (2021–2024), the most effective method for seed dormancy release and the production of two-year-old nursery plants of *P. peregrina* has been successfully developed.

The ability to produce more plants in less time, via nursery plants, is one of the key findings that result from this study. In addition, nursery plant production has important implications for conserving genetic diversity and supporting restoration efforts for native *P. peregrina* populations, as well as ensuring the success of its cultivation. It may be helpful to plant growers who wish to establish plantations with this protected, valuable, and medicinally significant plant species.

## Figures and Tables

**Table 1:** Properties of *Paeonia peregrina* seeds originating from various localities in Serbia. Seed mass data are reported as the mean ± standard error (n = 3). Differences were evaluated by one-way analysis of variance (ANOVA) completed with Duncan’s test (*p* ≤ 0.05). Different letters (a,b) indicate significant differences (*p* ≤ 0.05).

Locality (Seed Origin)	Average Number of Seed per Plant	Length (mm)	Width (mm)	Seed Mass (g)	Undeveloped Seeds (%)
Bogovo Guvno	56.71 ± 22.46	7.76 ± 0.43	5.11 ± 0.32	0.127 ± 0.028 a	3.3
Krivi vir	81.42 ± 33.53	7.87 ± 0.36	6.11 ± 0.44	0.141 ± 0.024 a	4.5
Pirot	25.00 ± 13.53	7.79 ± 0.41	5.40 ± 0.34	0.130 ± 0.037 a	3.8
Golina	33.90 ± 12.99	7.89 ± 0.34	6.20 ± 0.48	0.142 ± 0.023 a	3.1
Institute’s collection-Pančevo	50.91 ± 25.57	9.22 ± 0.65	6.60 ± 0.63	0.211 ± 0.025 b	2.9

**Table 2:** *P. peregrina* seed imbibition rate (%) based on the initial (fresh) seed weight and the applied GA_3_ concentration and imbibition period. Differences were evaluated by one-way analysis of variance (ANOVA) completed with Duncan’s test (*p* ≤ 0.05). Different letters (a–c) indicate significant differences (*p* ≤ 0.05).

Imbibition Period (h)	Imbibition Rate (%)			Control
	GA_3_ 200 mg L^−1^	GA_3_ 300 mg L^−1^	GA_3_ 400 mg L^−1^	
24	19.40 a	19.38 a	19.06 a	18.69 a
48	11.68 b	11.45 b	11.34 b	11.35 b
72	4.20 c	3.91 c	3.90 c	3.76 c

**Table 3:** Length of embryo (E) and endosperm (S) and their ratio (E:S ratio) during the germination of *P. peregrina* seeds. Data are reported as the mean ± standard error (n = 3). Differences were evaluated by one-way analysis of variance (ANOVA) completed with Duncan’s test (*p* ≤ 0.05). Different letters (a–c) indicate significant differences (*p* ≤ 0.05).

Seed Stage Description		Length (mm)		E:S Ratio
		Embryo (E)	Endosperm (S)	
Fresh seed	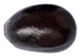	1.584 ± 0.35	7.50 ± 1.585	0.214 ± 0.031
Seed coat rapture	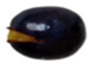	3.32 ± 0.27	9.42 ± 1.82	0.36 ± 0.06
Radicle emergence	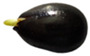	6,15 ± 0.96	9.61 ± 1.3	0.64 ± 0.04

**Table 4:** Altitude, latitude, and longitude of natural habitats of *Paeonia peregrina* and the Institute’s collection.

Locality	Elevation (m.a.s.l.)	Latitude	Longitude	Vaucher no (BUNS *)
Bogovo guvno	952	43°33′ N	21°46′ E	2-674
Krivi vir	467	43°49′ N	21°46′ E	2-675
Pirot	666	43°07′ N	22°27′ E	2-679
Golina	299	43°46′ N	22°19′ E	2-673
Institute’s collection, Pančevo	74	44°52′ N	20°42′ E	

* BUNS.

## Data Availability

Data are contained within the article.
